# A rare event of late agranulocytosis during clozapine use in schizophrenia and the importance of monitoring vitamin B12 levels in patients with severe psychiatric conditions: a case report

**DOI:** 10.47626/1516-4446-2024-3852

**Published:** 2024-11-25

**Authors:** Alessandra Faggion Mocellin, Clara de Oliveira Lapa, Clarissa Severino Gama

**Affiliations:** 1 Hospital Psiquiátrico São Pedro, Porto Alegre, RS, Brazil; 2 Hospital de Clínicas de Porto Alegre, Porto Alegre, RS, Brazil

Agranulocytosis is a severe event described in clozapine users, especially in the first 18 weeks of treatment.^1^ After this period, this outcome is extremely uncommon, especially in those who maintain an absolute neutrophil count (ANC) > 2,000/μL during the first year of use.^1^ Therefore, other associations that could contribute to a decreased neutrophil count should be investigated. We report a case of neutropenia in a 44-year-old man with schizophrenia who had been using clozapine for 12 years. The patient was admitted to a psychiatric hospital with an ANC of 680/μL and a B12 serum level of 172 ng/mL. Values > 300 ng/mL are considered normal, 300-190 ng/mL borderline, and < 190 ng/mL, deficient.^2^ Clozapine was discontinued, and a comprehensive hematological evaluation was performed. The only significant abnormality was the low B12 level. The patient received intramuscular B12, haloperidol, and was stimulated with filgrastim. The day after filgrastim was administered, the ANC increased to 2,010/uL. After 1 week, clozapine was reintroduced and haloperidol was withdrawn. The patient was discharged 3 weeks after admission with an ANC of 3,146/μL, and a B12 level of 1,202 ng/mL. He has maintained an ANC > 2,000/μL and serum B12 within normal limits 12 months after hospital discharge. Considering his prolonged clozapine use without previous adverse hematological events, other factors that could lead to reduced ANC require investigation. B12 deficiency stands out, which can interfere with the production of hematological lineages.^3^ Neutropenia due to vitamin B12 deficiency is a rare and potentially fatal event.^4^ Severe mental disorders are often correlated with hypovitaminosis, which can cause various symptoms, treatment resistance, or clinical deterioration.^5^ Schizophrenia is a risk factor for vitamin B12 deficiency and, despite its high prevalence, it is rarely evaluated in routine examinations.^6^ Clozapine is an effective antipsychotic for treatment-resistant schizophrenia, but its potential hematological adverse effects^1^ often limit its use. The relationship between clozapine and neutropenia is well-documented,^1^ but the effects of nutritional deficiencies, such as vitamin B12 deficiency, may be underestimated in patients with severe psychiatric disorders, whether they use clozapine or not.^5^,^6^ Vitamin B12 is essential for DNA synthesis and blood cell production. Its deficiency can not only cause neutropenia but also exacerbate psychiatric symptoms.^5^ Moreover, low levels of vitamin B12 are associated with worse prognosis in patients with schizophrenia.^6^ The aim of this report is not to demonstrate that low serum B12 can increase the risk of clozapine-induced neutropenia, but rather to raise awareness that patients with severe psychiatric disorders tend to have low serum B12 levels, which can pose a serious life-threatening risk in various domains. Detecting and correcting serum B12 levels may be a valuable and cost-effective strategy, not only for clozapine users but for the whole population with severe psychiatric disorders, given the association with worsening psychiatric symptoms, treatment resistance, and clinical deterioration.

## Disclosure

The authors report no conflicts of interest.
